# Supplementary material for: ITLN1 modulates invasive potential and metabolic reprogramming of ovarian cancer cells in omental microenvironment
Source: Nat Commun. 2020 Jul 15;11:3546. doi: 10.1038/s41467-020-17383-2 (PMC7363861; doi:10.1038/s41467-020-17383-2)
Supplement: Supplementary file 1 — Supplementary Information [file 41467_2020_17383_MOESM1_ESM.pdf]

Supplementary Information

ITLN1 modulates invasive potential and metabolic reprogramming of ovarian cancer cells in omental microenvironment

Au-Yeung et al.

Supplementary Fig. 1.

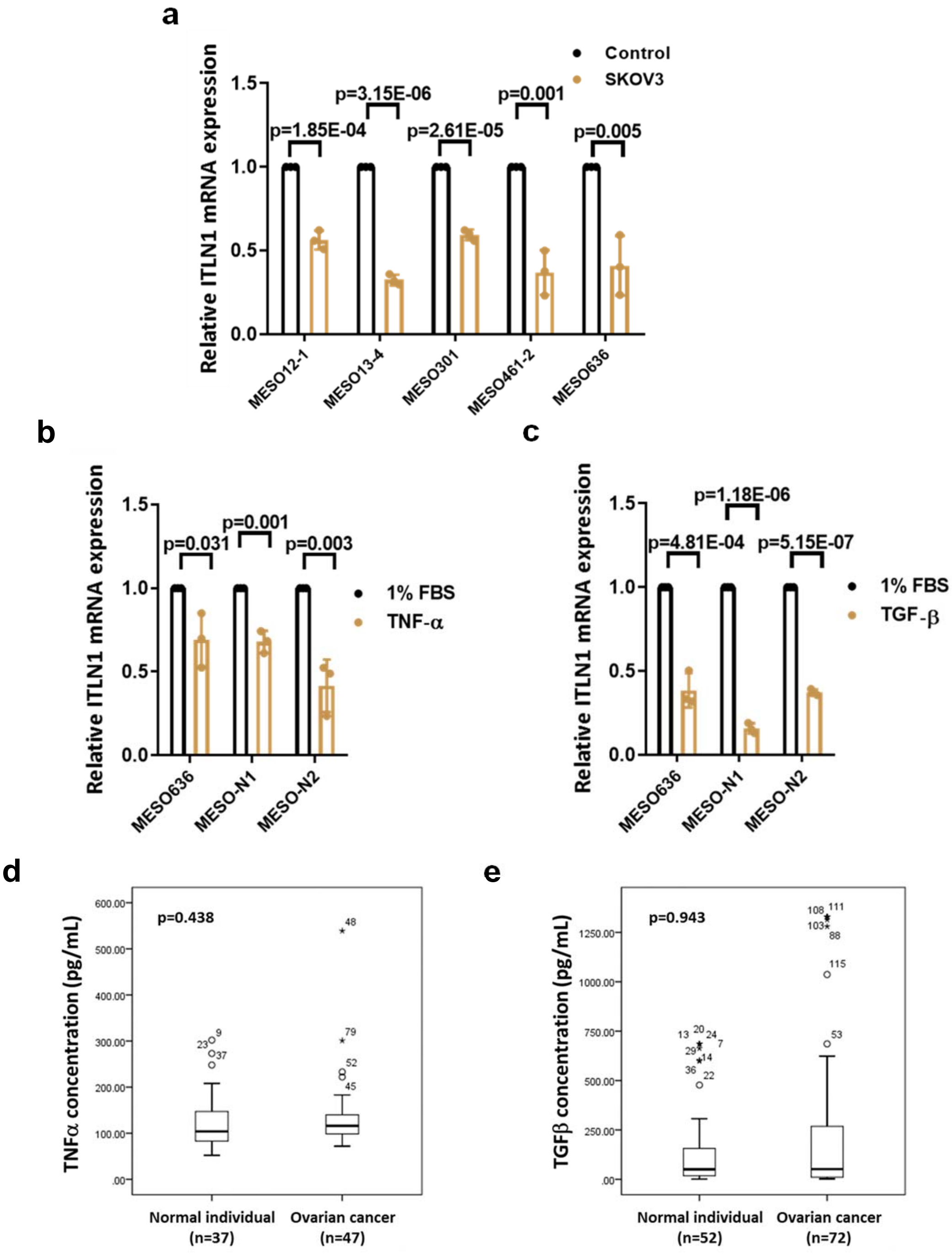

Supplementary Fig. 1: Proinflammatory cytokines regulate ITLN1. (**a**)-(**c**) QRT-PCR analysis shows (**a**) that in normal mesothelial cells co-cultured with SKOV3 had lower ITLN1 mRNA levels than did mesothelial cells cultured without SKOV3; (**b**) a lower ITLN1 mRNA level in normal mesothelial cells treated with TNF-α compared to untreated controls. Cells were treated with 10 ng/mL TNF-α for 24 h in medium supplied with 1% FBS before total RNA extraction; (**c**) a lower ITLN1 mRNA level in normal mesothelial cells treated with TGF-β for 24 h compared to untreated controls. Cells were treated with 5 ng/mL TGF-β for 24 h in medium supplied with 1% FBS before total RNA extraction. Results from three independent experiments were averaged and are shown as mean±SD (two-tailed t-test). FBS=fetal bovine serum. (**d**)-(**e**) In the box plot, the boxes represent the interquartile range of the records, and the lines across the boxes indicate the median value of the records. The whiskers indicate the highest and lowest values among the records that are no more than 1.5 times greater than the interquartile range.

Supplementary Fig. 2.

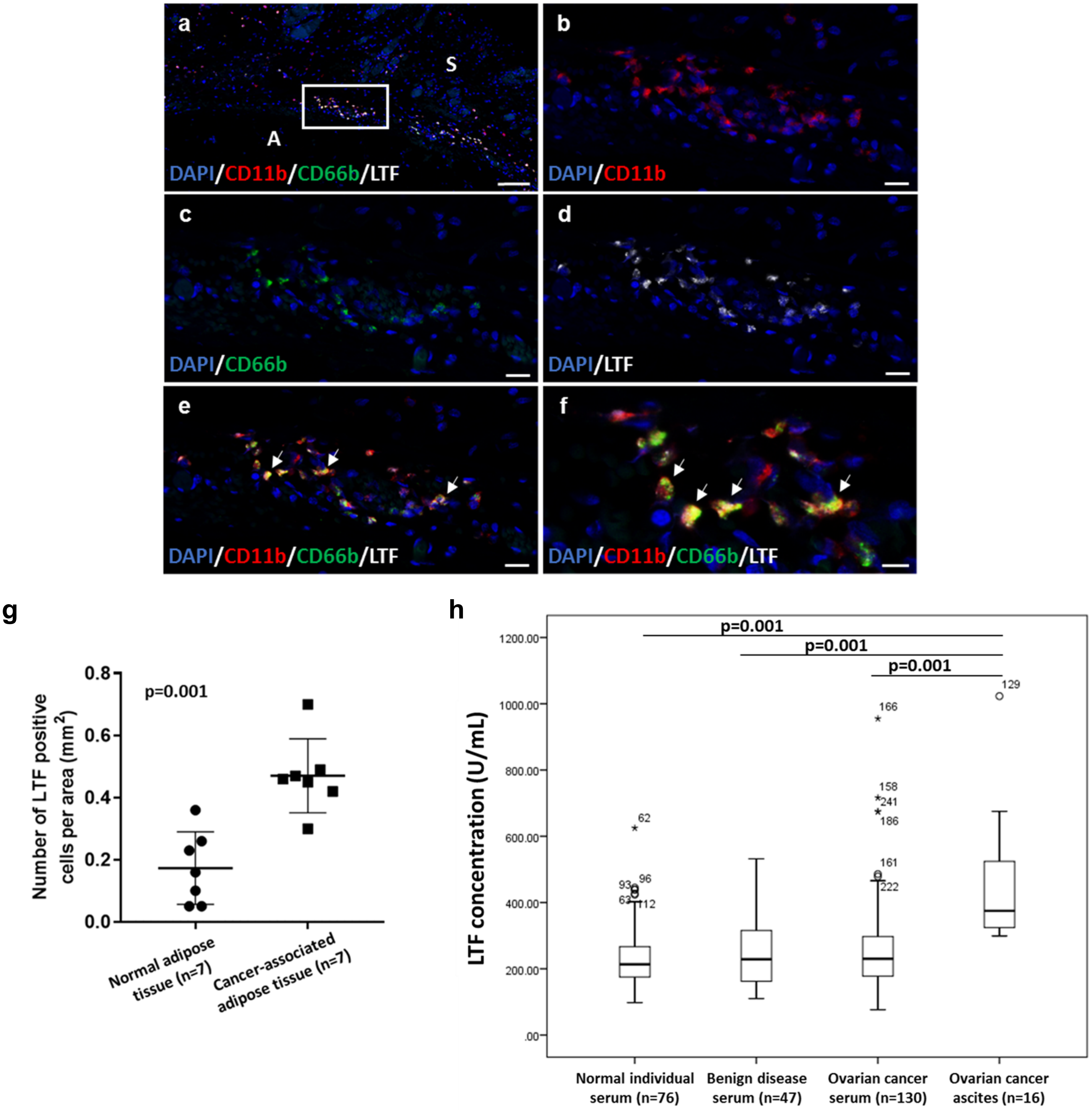

Supplementary Fig. 2: LTF is predominantly expressed in neutrophils and overexpressed in cancer-associated adipose tissues. (**a**)-(**f**) LTF is immunolocalized on FFPE sections of omental adipose tissue from HGSC patients together with CD11b and CD66b, markers for neutrophils using Opal multiplex immunohistrochsmistry. (**e**)-(**f**) LTF is co-localized with CD11b and CD66b (arrows). Representative microscopic images are shown. N=3. Bar= 100µm (**a**), 20µm (**b**)-(**e**) and 10µm (**f**). A=Adipocytes; S=Stroma. (**g**) The expression level of LTF is significantly higher in cancer-associated adipose tissue from HGSC patients than in normal adipose tissue from healthy women (p=0.001; Mann-Whitney u test; n=7 in both groups). (**h**) There is no significant difference in circulating level of LTF in sera from healthy women (n=76), patients with benign diseases (n=47) and HGSC patients (n=130). The level of LTF in ascites from HGSC patients (n=16) is significantly upregulated compared to those in sera (p=0.001; Mann-Whitney U test). In the box plot, the boxes represent the interquartile range of the records, and the lines across the boxes indicate the median value of the records. The whiskers indicate the highest and lowest values among the records that are no more than 1.5 times greater than the interquartile range.

Supplementary Fig. 3.

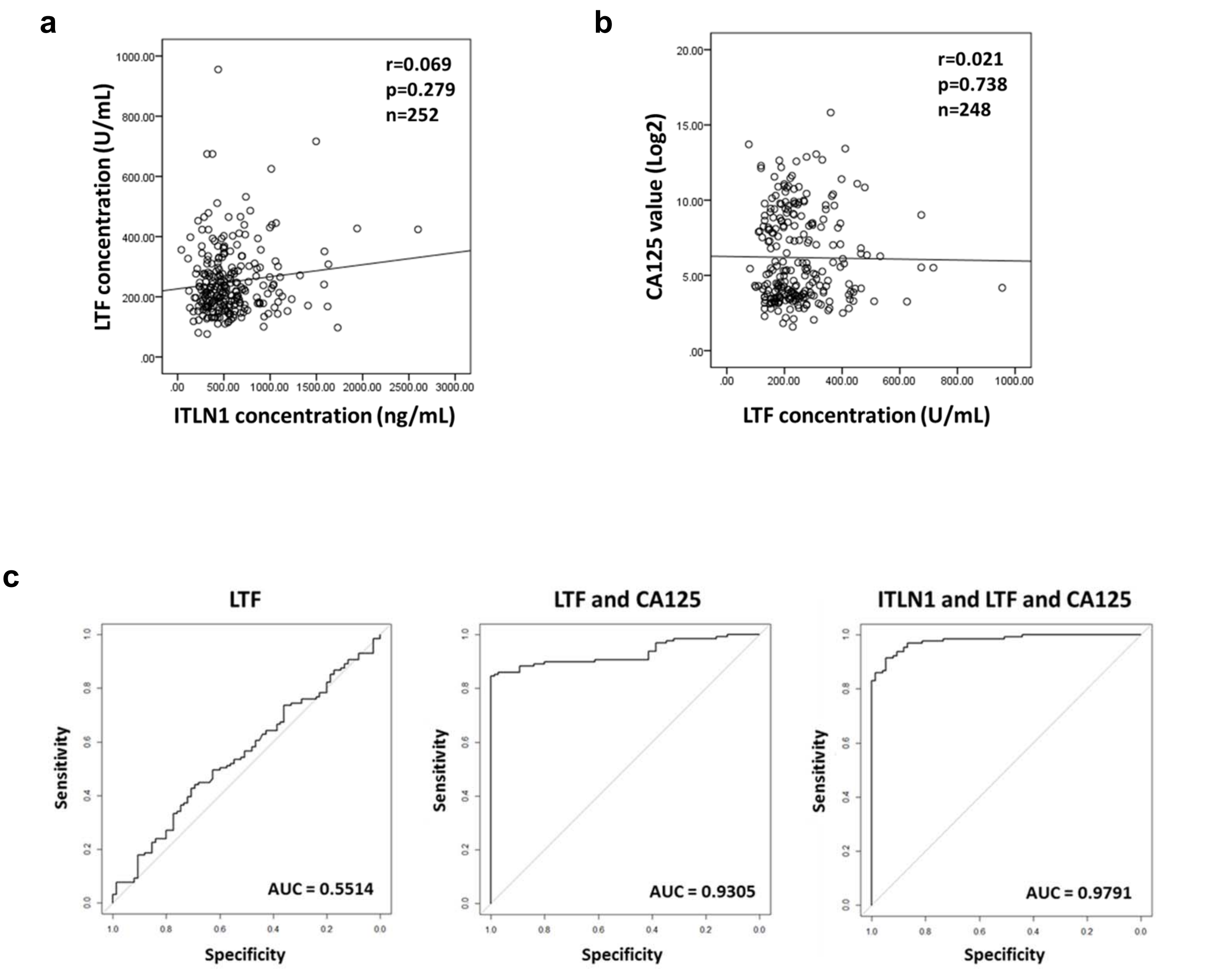

Supplementary Fig. 3: . (**a**)-(**b**) Graph shows no significant correlation (two-tailed t-test) between LTF (**a**) and ITLN1, (**b**) and CA125 levels in serum collected from normal women, patients with benign gynecologic disease, and HGSC patients. (**c**) Receiver operating characteristic curves show a significantly smaller area under the curve (AUC) for LTF alone than CA125 alone (p=2.2E-16), and that for LTF with CA125 does not have a significant larger AUC than CA125 alone (p=0.448). However, that for LTF in combination with ITLN1 and CA125 showed a significant larger AUC than CA125 alone (p=0.005; one-tailed ROC.test function) or CA125 with ITLN1 (p=0.037; one-tailed ROC.test function) (Fig. 2e).

Supplementary Fig. 4.

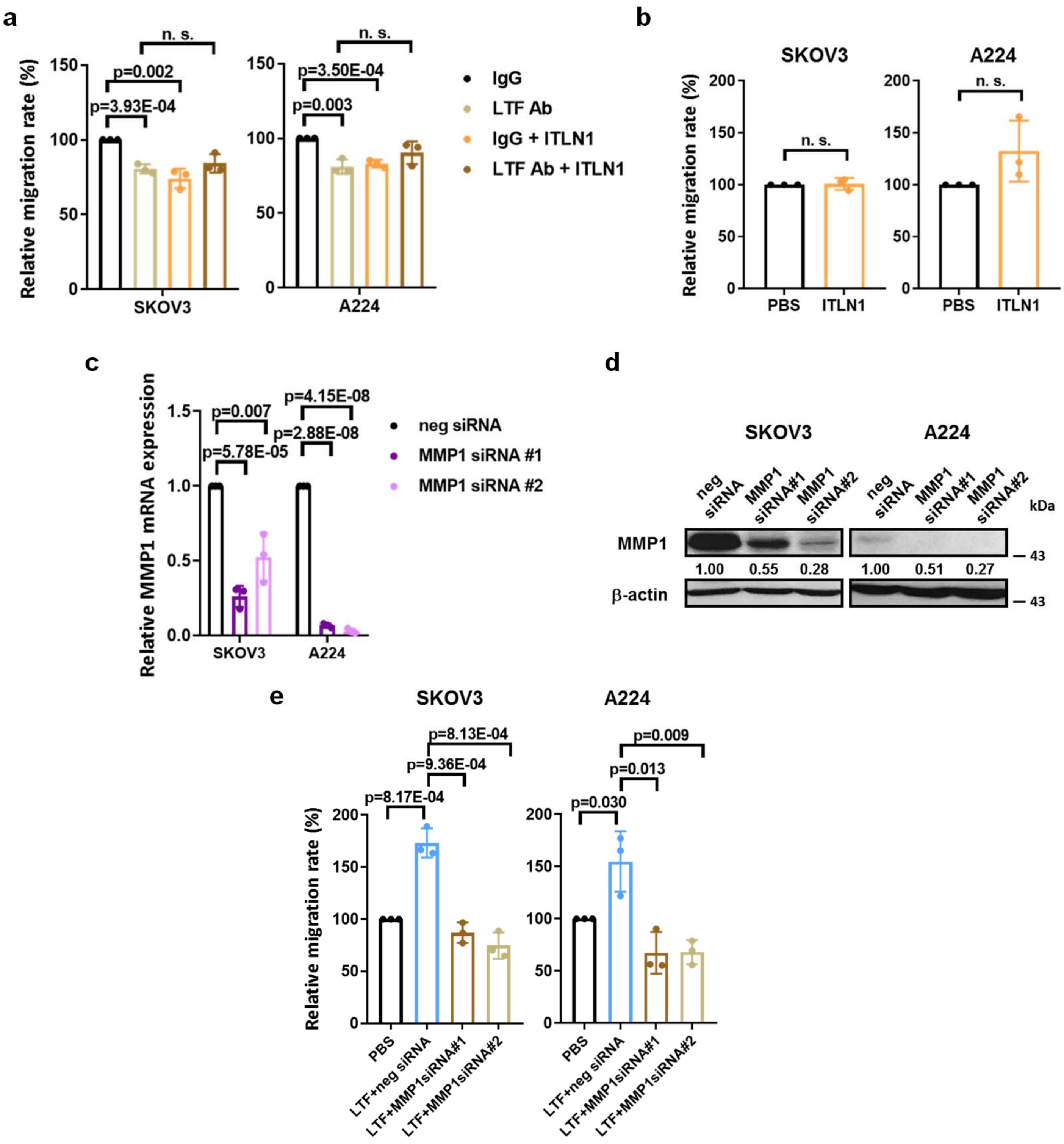

Supplementary Fig. 4: ITLN1 opposes the motility-promoting effect of LTF. ITLN1 does not suppress cell migration ability in SKOV3 and A224 (**a**) in LTF-depleted culture medium. Cells were treated with LTF antibody to deplete LTF in culture medium with FBS before ITLN1 treatment. IgG was used as a control; (**b**) in FBS-free culture medium. Results from three independent experiments were averaged and are shown as mean±SD (two-tailed t-test). n.s.=not significant (p>0.05). (**c**)-(**d**) The effect of MMP1-specific siRNAs on (**c**) mRNA and (**d**) protein levels in SKOV3 and A224 cells. Results from three independent experiments were averaged and are shown as mean±SD. β-actin served as a loading control. Relative normalized protein levels with respect to the corresponding control are presented. (**e**) LTF induces cell migration ability in SKOV3 and A224 compared to control cells, while MMP1 siRNAs counteracted the effect of LTF on cell migration. Results, presented as mean±SD (two-tailed t-test), shows the average from three independent experiments with duplicated samples.

Supplementary Fig. 5.

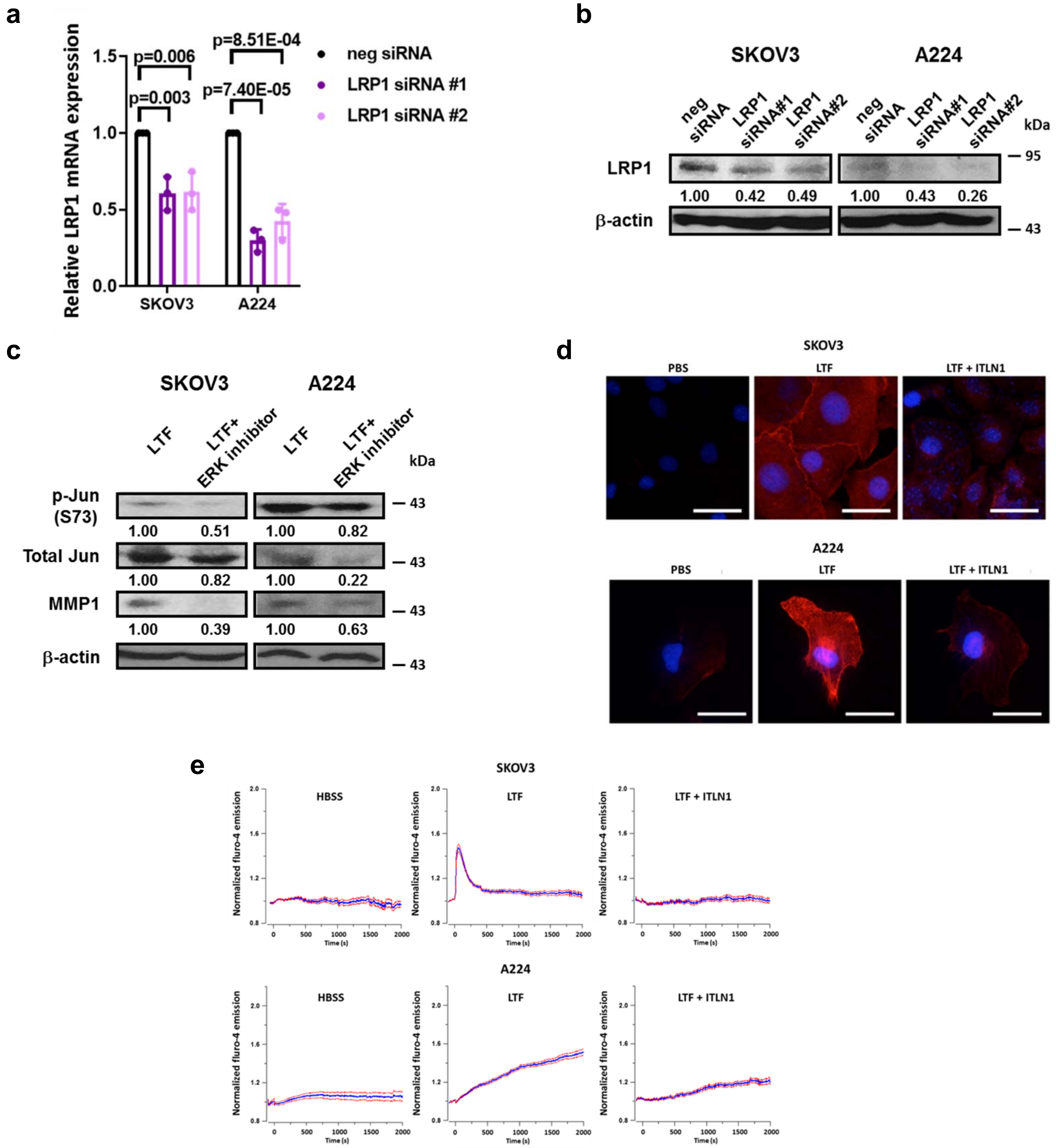

Supplementary Fig. 5: ITLN1 inhibits the LTF/LRP1/MMP1 signaling pathway and suppresses LTF-modulated calcium mobilization and stress fiber formation. (**a**)-(**b**) The effect of LRP1-specific siRNAs on (**a**) mRNA and (**b**) protein levels in SKOV3 and A224 cells. Results from three independent experiments were averaged and are shown as mean±SD (two-tailed t-test). β-actin served as a loading control. Relative normalized protein levels with respect to the corresponding control are presented. (**c**) Western blot analyses show lower protein levels of p-Jun (S73), total Jun, and MMP1 in LTF-treated SKOV3 and A224 with 10 μM ERK inhibitor (FR180204) compared to control cells without ERK inhibitor. β-actin served as a loading control. Relative normalized protein levels with respect to the corresponding control are presented. Three independent experiments were performed. (**d**) Representative microscopic images show that LTF activated stress fiber formation in SKOV3 and A224, while ITLN1 abrogated the activation. Bar=5μm. (**e**) Graphs show the mean normalized time course of LTF-induced Ca2+ mobilization in SKOV3 and A224; ITLN1 abrogated the induction. Data is shown as mean ± the standard error of the mean; n=4; HBSS=Hank's balanced salt solution.

Supplementary Fig. 6.

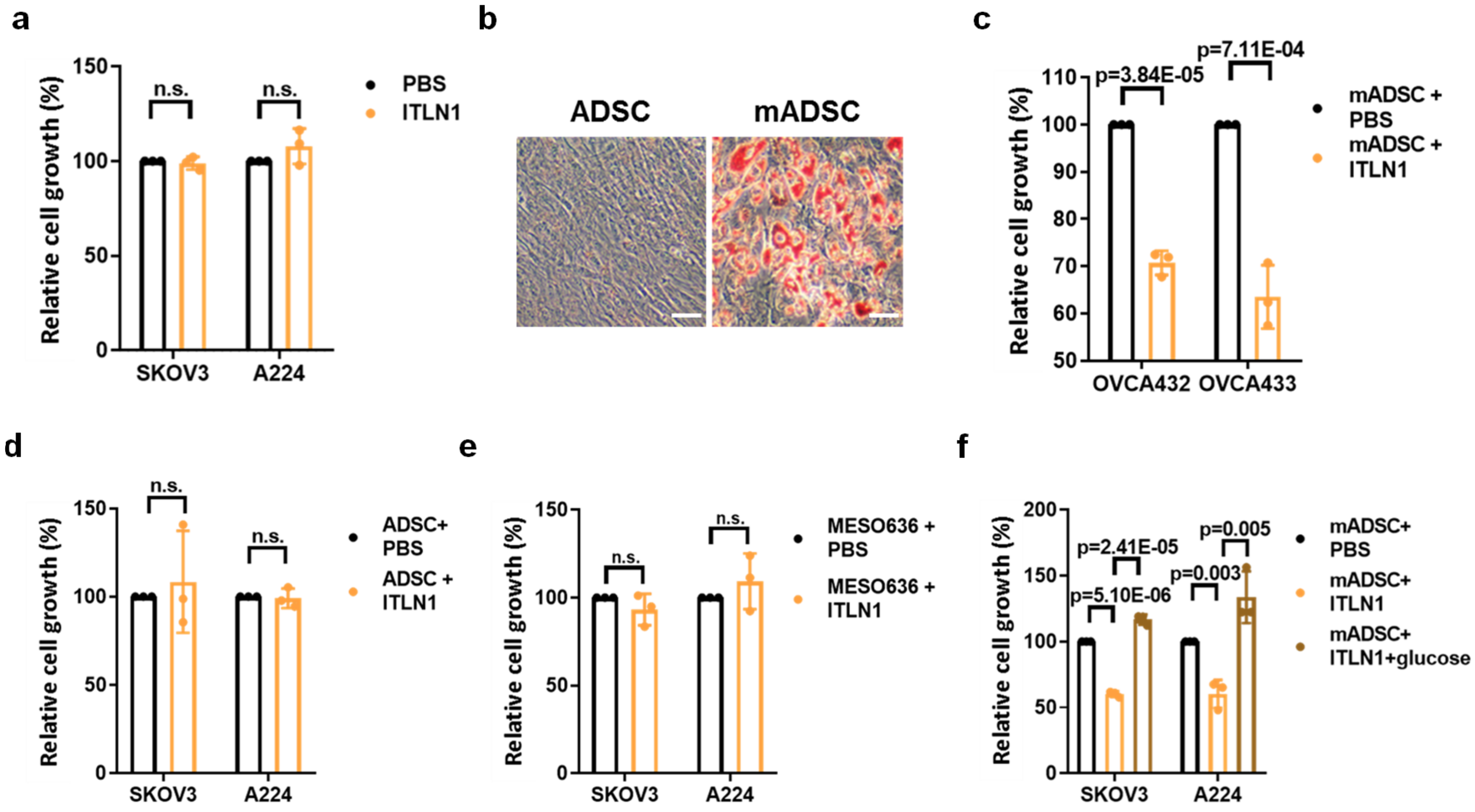

Supplementary Fig. 6: ITLN1 suppresses ovarian cancer cell growth in the presence of mature adipocytes. (**a**) ITLN1 has no significant effect on the growth of SKOV3 and A224. Cells were treated with 500 ng/mL ITLN1 for 72 h. Cell growth was measured by MTT assay. (**b**) Preadipocytes (ADSC) and mADSC were characterized using Oil Red O staining. Representative images were shown. Bar=50μm. (**c**) ITLN1 suppresses OVCA432 and OVCA433 cell growth in the presence of mature adipocytes (mADSC). Cells were co-cultured with ITLN1-treated mature adipocytes for 72 h. (**d**)-(**e**) ITLN1 has no significant effect on the growth of SKOV3 and A224 in the presence of (**d**) preadipocytes and (**e**) mesothelial cells (MESO636). Cells were co-cultured with ITLN1-treated preadipocytes and mesothelial cells, respectively for 72 h. (**f**) Addition of 5.5mM glucose counteracts the effect of ITLN1-treated mature adipocytes on cell growth in SKOV3 and A224. Results in bar charts, presented as mean±SD (two-tailed t-test), show the average from three independent experiments. n.s.=not significant (p>0.05).

Supplementary Fig. 7.

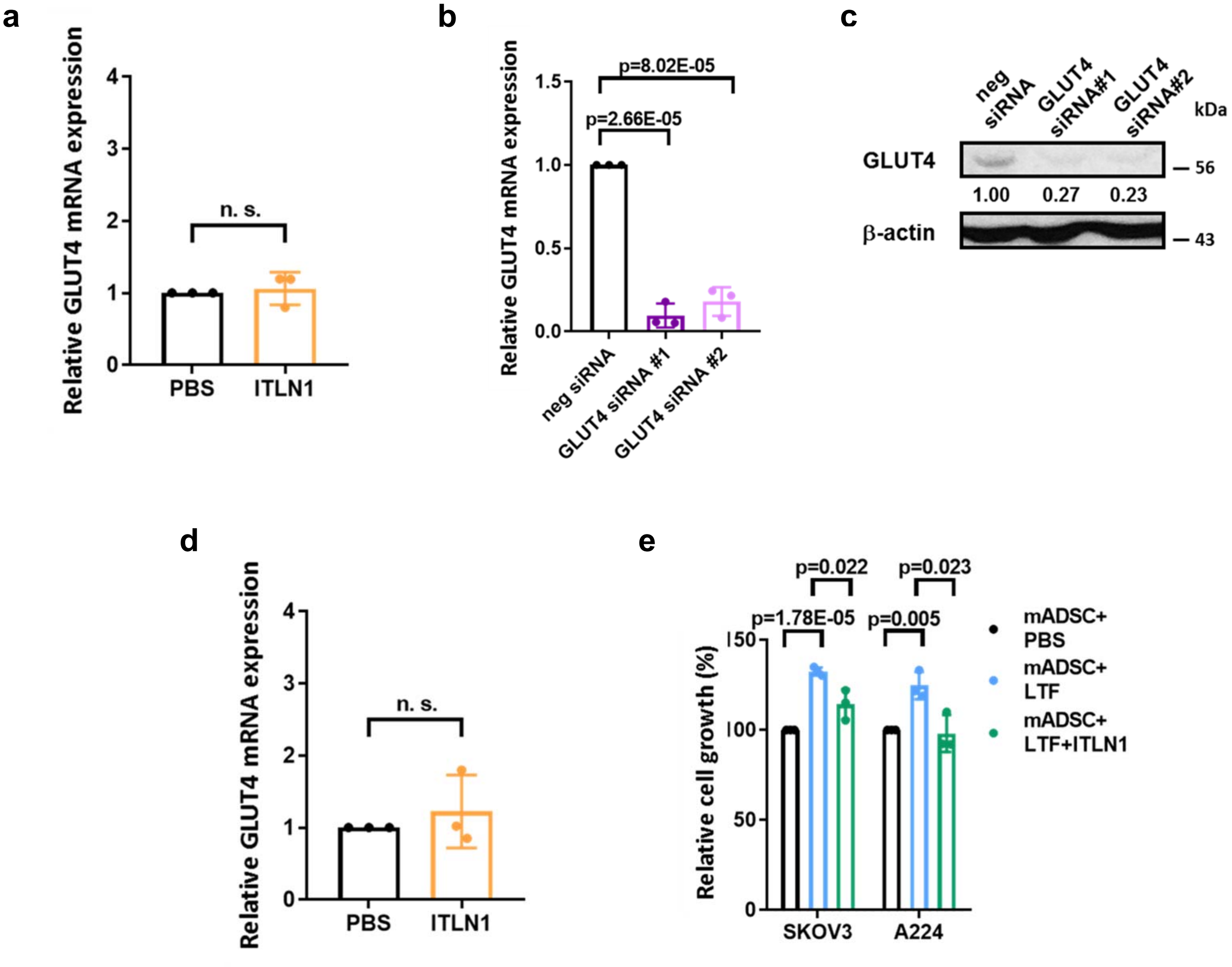

Supplementary Fig. 7: ITLN1 counters LTF effects on ovarian cancer cell growth via GLUT4. (**a**) QRT-PCR analysis shows the relative GLUT4 mRNA expression in ITLN1-treated mADSC compared to control cells in the absence of insulin. (**b**) QRT-PCR analysis shows the relative GLUT4 mRNA expression in mADSC transfected with two GLUT4-specific siRNAs and control siRNA. (**c**) Western blot analysis shows GLUT4 protein expression in mADSC transfected with two GLUT4-specific siRNAs and control siRNA. β-actin was served as a loading control. Relative normalized protein levels with respect to the corresponding control are presented. Three independent experiments were performed. (**d**) QRT-PCR analysis shows the relative GLUT4 mRNA expression in ITLN1-treated mADSC compared to control cells in FBS-free culture medium in the presence of insulin. (**e**) LTF induces SKOV3 and A224 cell growth in the presence of mADSC, while ITLN1 counteracted the effect. Cells were co-cultured with ITLN1 and/or LTF-treated mature adipocytes for 72 h. Results in bar charts, presented as mean±SD (two-tailed t-test), show the average from three independent experiments. n.s.=not significant ($p>0.05$).

Supplementary Fig. 8.

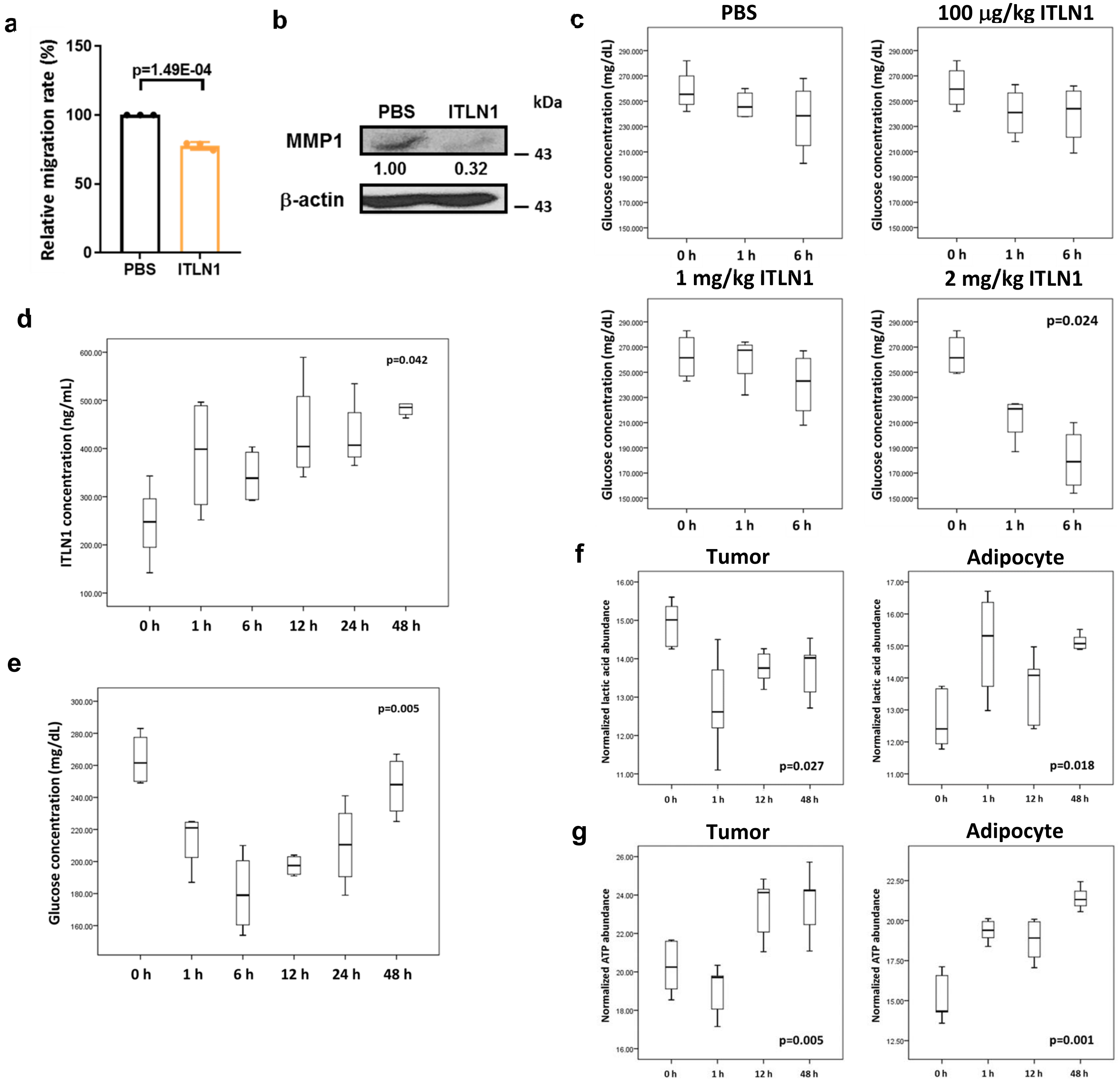

Supplementary Fig. 8: ITLN1 induces metabolic shift in ovarian cancer cells *in vivo*. (**a**) Recombinant mouse ITLN1 protein suppresses cell migration ability in IG10 compared to control cells *in vitro*. Results, presented as mean±SD (two-tailed t-test), show the average from three independent experiments. (**b**) Western blot analysis shows a lower MMP1 protein level in ITLN1-treated IG10 compared to control cells. β-actin was served as a loading control. Relative normalized protein levels with respect to the corresponding control are presented. Three independent experiments were performed. (**c**) Box plots shows a significantly lower glucose concentration in serum collected at different time points from IG10 cell-bearing C57BL/6 mice with PBS and ITLN1 injection (100 μg/kg, 1 mg/kg, and 2 mg/kg; n=5 in each group; p=0.024; Kruskal-Wallis H test). (**d**)-(**e**) Box plot shows a significantly (**d**) higher ITLN1 concentration and (**e**) lower glucose concentration in serum collected at different time points from IG10 cell-bearing C57BL/6 mice with 2 mg/kg ITLN1 injection (n=5 in each group; p=0.042 and 0.005, respectively; Kruskal-Wallis H test). (**f**)-(**g**) Box plot shows the normalized (**f**) lactic acid abundance (ion adduct: [M-H]; m/z: 89.0244) and (**g**) ATP abundance (ion adduct: [M-H]; m/z: 505.9885) in tumor cells and adipocytes at different time points after ITLN1 was intraperitoneally injected into C57BL/6 mice (n=5 in each group; p=0.027 and p=0.018, respectively; p=0.005 and p=0.001, respectively; Kruskal-Wallis H test). (**c**)-(**g**) In the box plot, the boxes represent the interquartile range of the records, and the lines across the boxes indicate the median value of the records. The whiskers indicate the highest and lowest values among the records that are no more than 1.5 times greater than the interquartile range.

Supplementary Fig. 9.

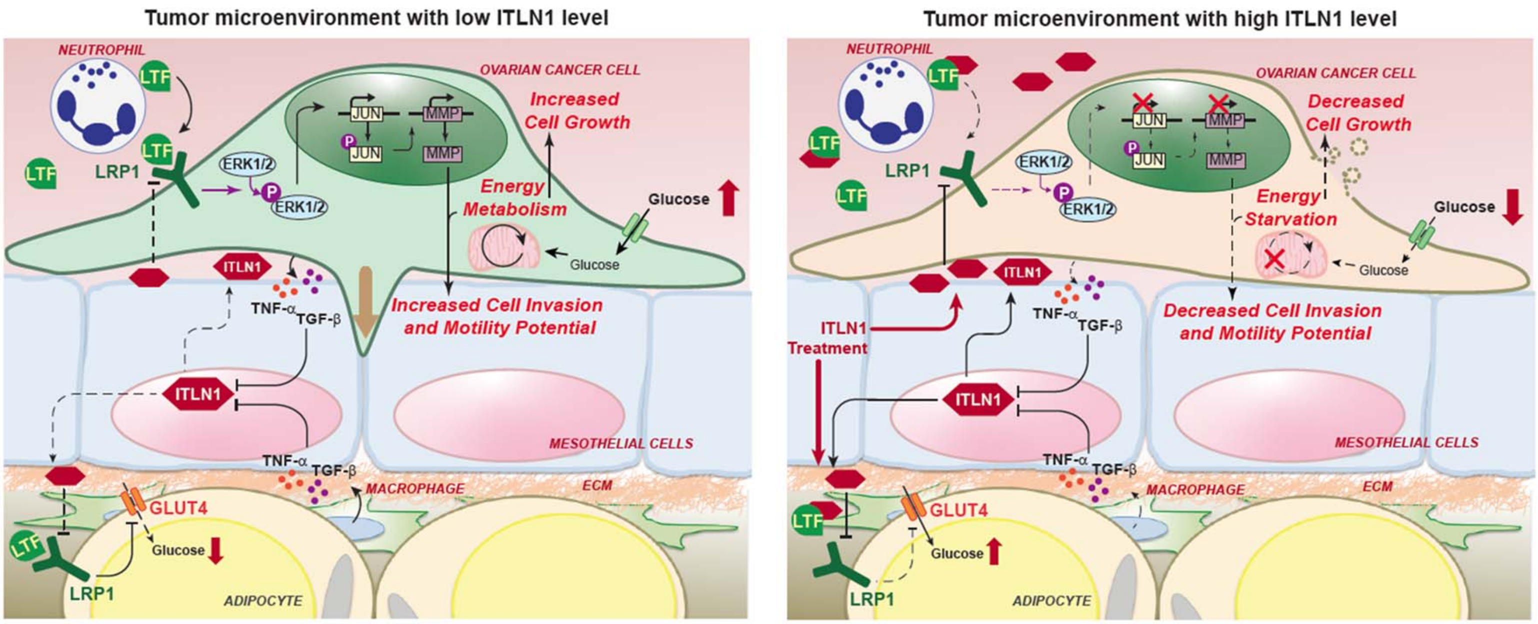

Supplementary Fig. 9: Schematic summarizes the roles of ITLN1 in ovarian cancer invasive potential and metabolic reprogramming in the omental microenvironment. Left Panel: In the tumor microenvironment with low ITLN1 level, the tumor-suppressive effect of ITLN1 are inhibited by TNF-α and TGF-β secreted by cancer cells and macrophages. This leaves circulating LTF to bind to LRP1 that activates downstream MMP1 signaling pathway leading to the higher metastatic propensities of the OC cells. In adipocytes, binding of LTF to LRP1 downregulates the expression of glucose transporter GLUT4. Further, OC cells have high glucose uptake and glycolytic phenotype that contributes to increased proliferation and metastatic properties. Right Panel: Treatment with ITLN1 binds to LTF, thereby interfering with the LTF-LRP1 binding and subsequent activation of MMP1. In adipocytes, ITLN1 also abrogates the LTF-LRP1 suppression of GLUT4 and increases their glucose uptake in an insulin-dependent manner. This increase in glucose uptake in adipocytes leads to glucose starvation in the OC cells, therefore, ITLN1 indirectly regulates the glycolytic phenotype of OC cells. Summarily, there effects lead to a reduction in the invasive potential and growth rate of OC cells. Solid lines: Strong regulation, Dashed lines: Weak regulation.

Supplementary Fig. 10.

Figure 3e

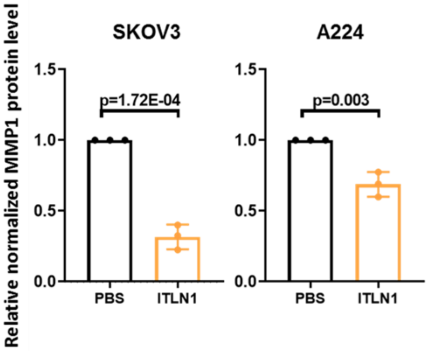

Figure 4f

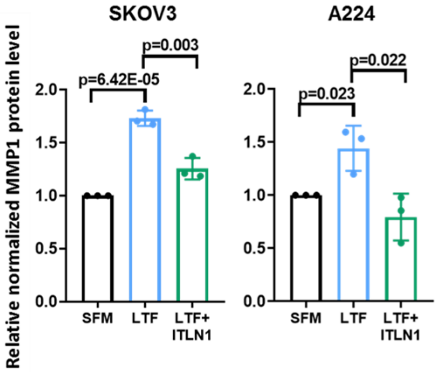

Figure 5a

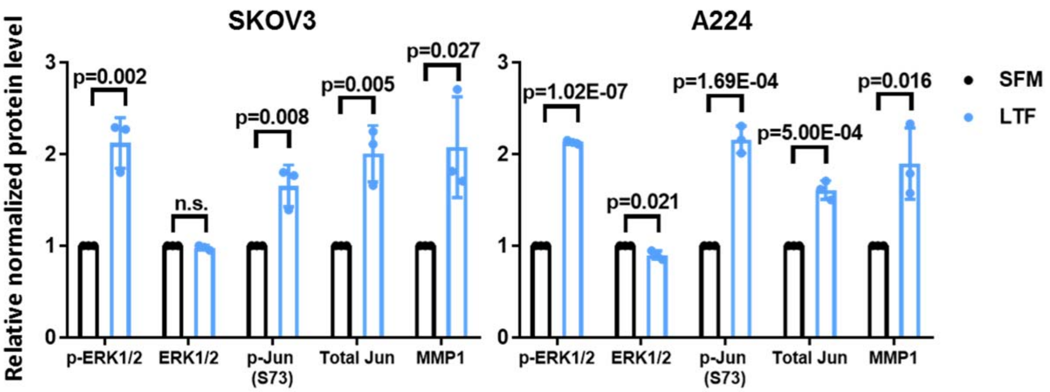

Figure 5b

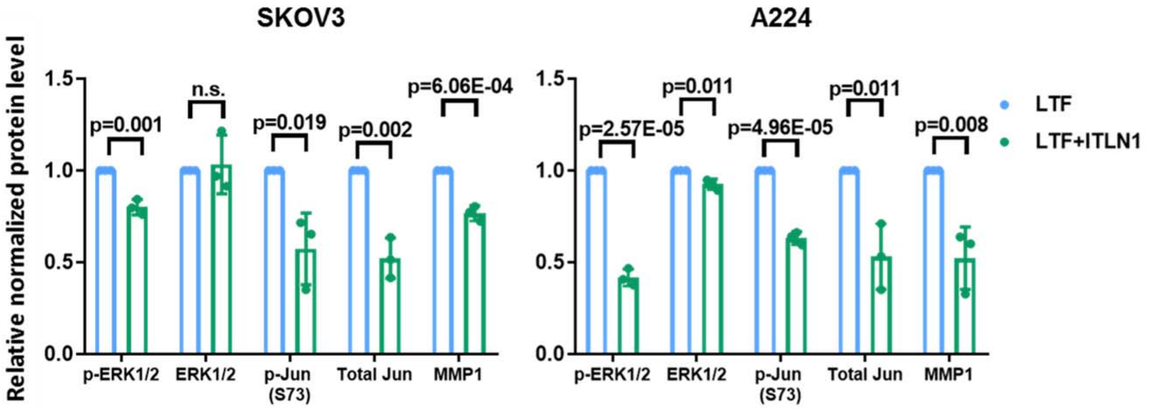

Figure 5c

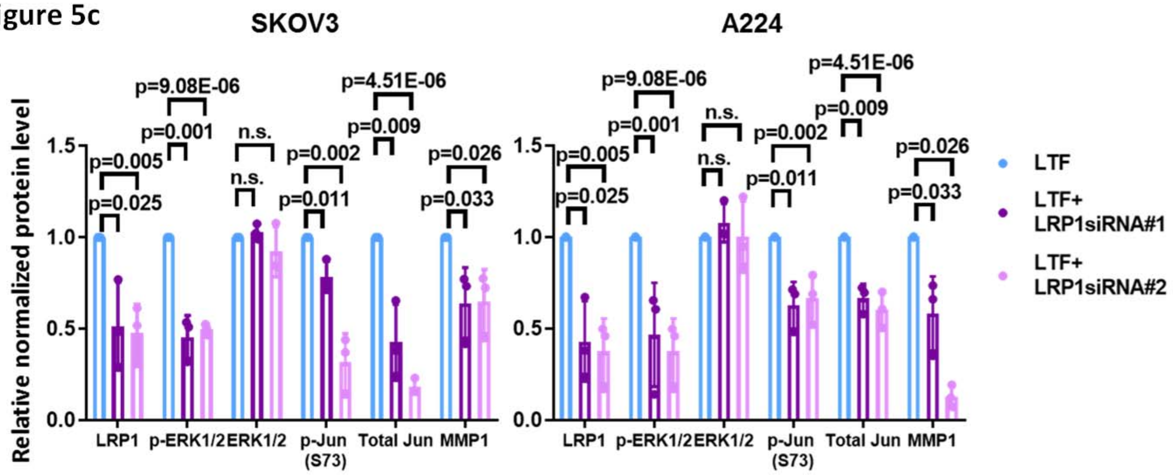

Figure 6d

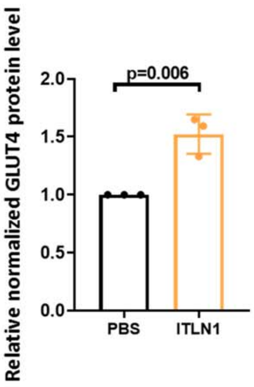

Supplementary Fig. 10: Quantification of Western blot analysis in Fig. 3-6. Results from three independent experiments were averaged and are shown as mean±SD (two-tailed t-test). n.s. = not significant (p>0.05).

Supplementary Fig. 11.

Supplementary Fig. 4d

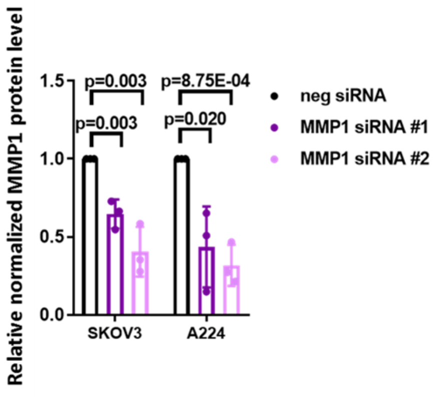

Supplementary Fig. 5b

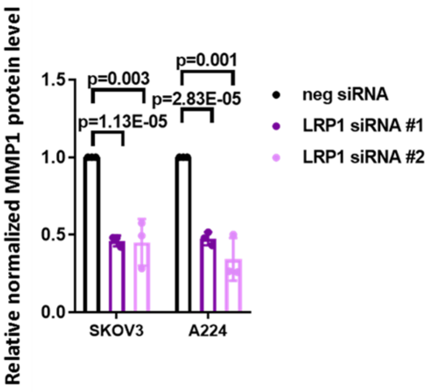

Supplementary Fig. 5c

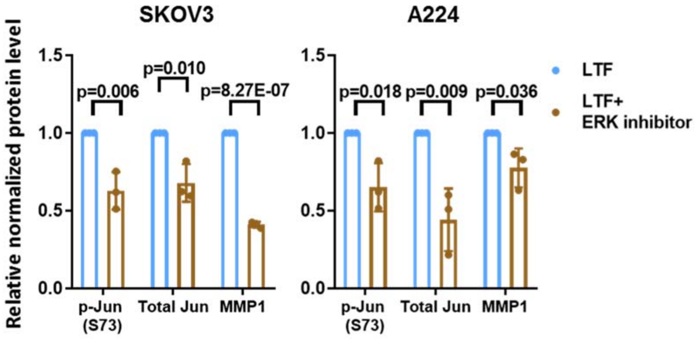

Supplementary Fig. 7c

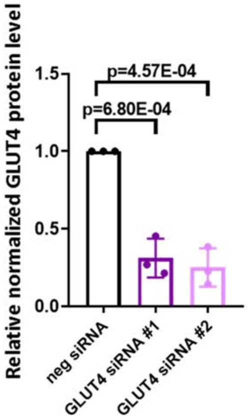

Supplementary Fig. 8b

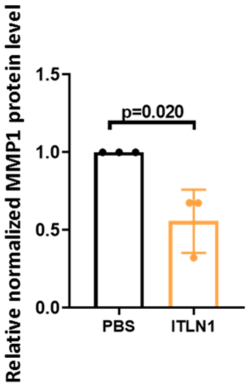

Supplementary Fig. 11: Quantification of Western blot analysis in Supplementary Fig. 2-5. Results from three independent experiments were averaged and are shown as mean±SD (two-tailed t-test).

Supplementary Fig. 12.

Fig. 1*d*

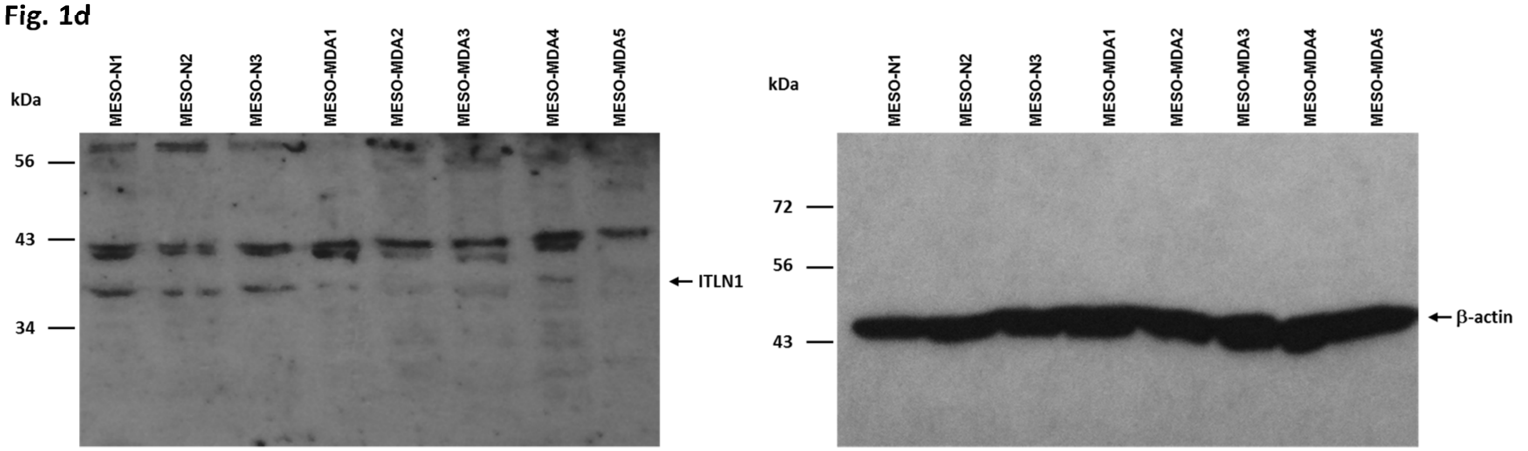

Fig. 3e

SKOV3

A224

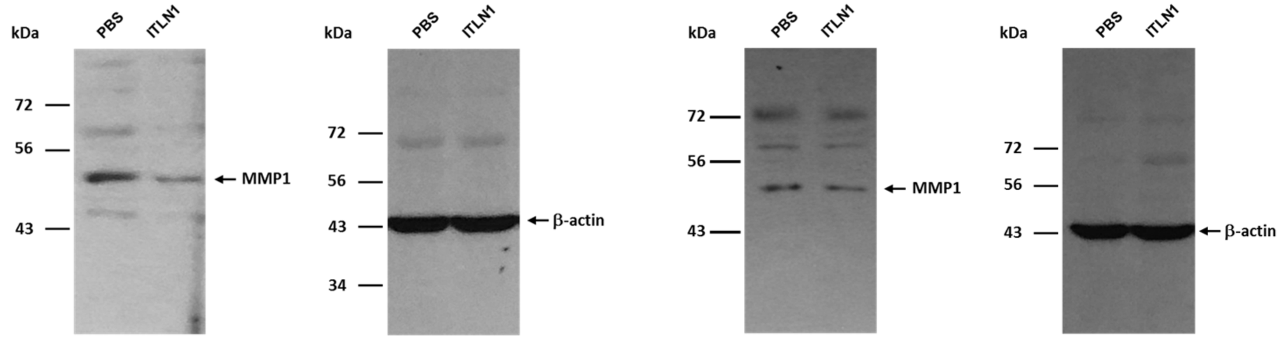

Fig. 4a

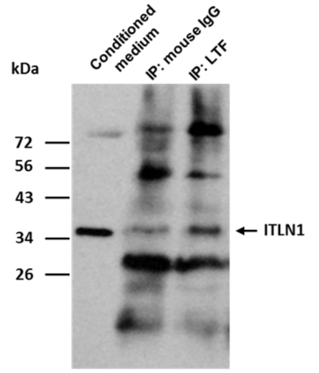

Fig. 4f

SKOV3

A224

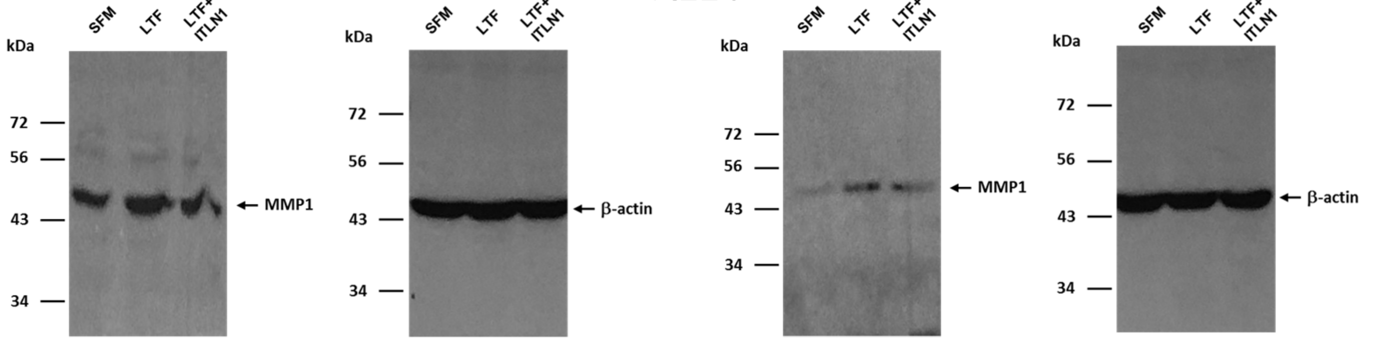

Supplementary Fig. 12: Original blots for Figs. 1-4.

Supplementary Fig. 13.

Fig. 5a

SKOV3

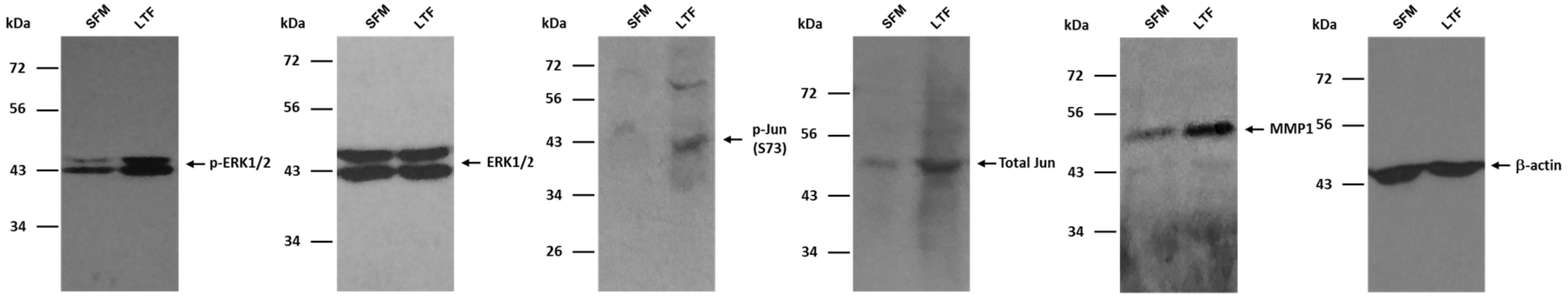

A224

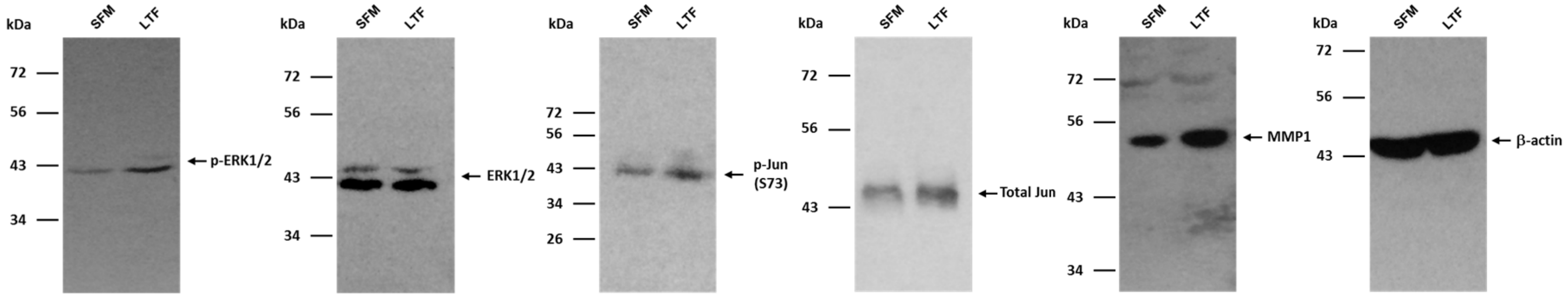

Fig. 5b

SKOV3

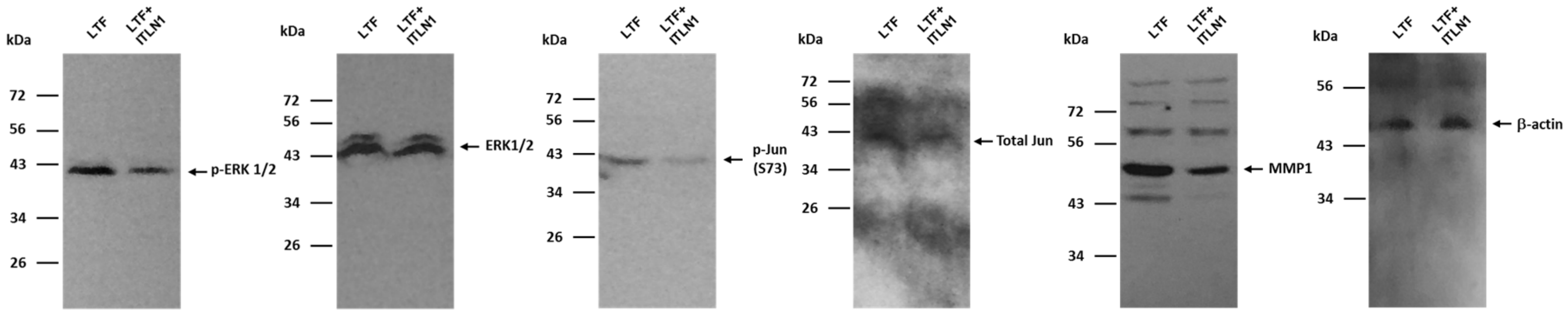

A224

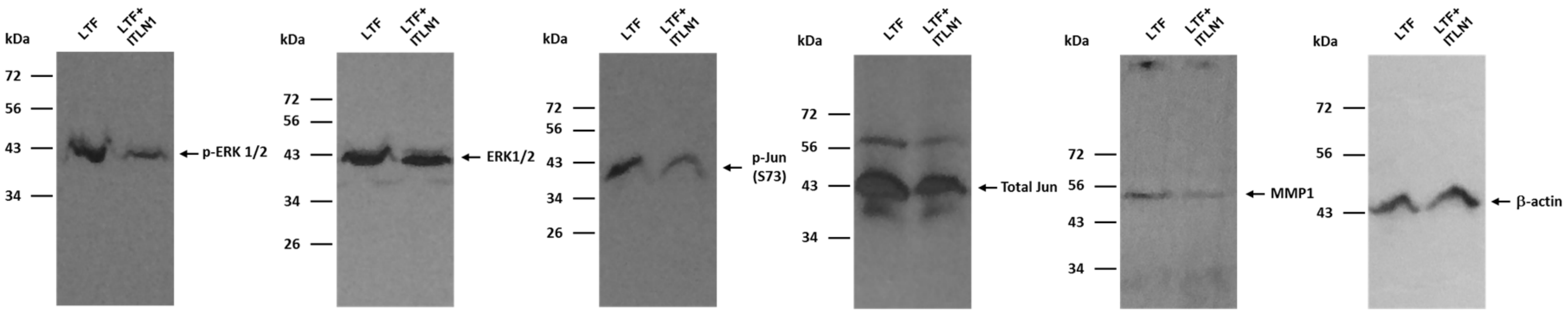

Supplementary Fig. 13: Original blots for Fig. 5a and b.

Supplementary Fig. 14.

Fig. 5c

SKOV3

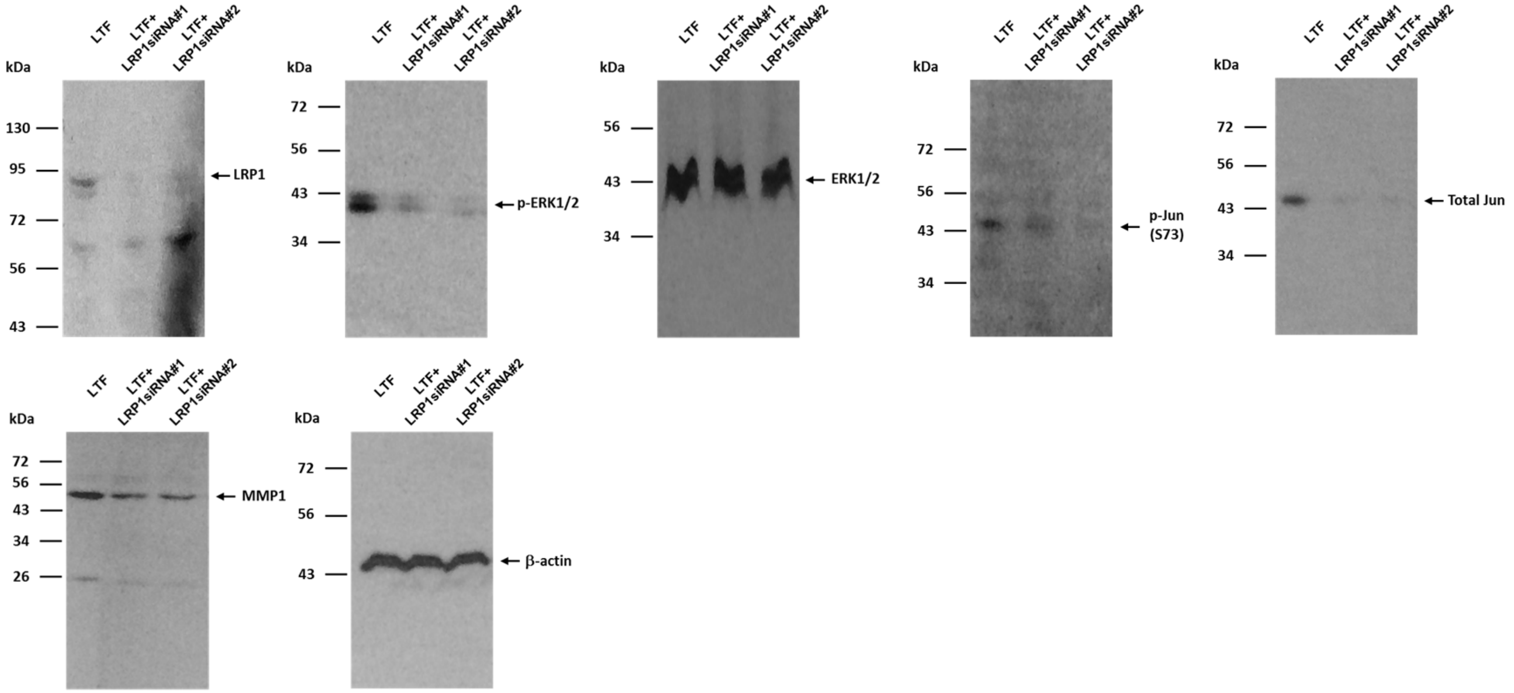

A224

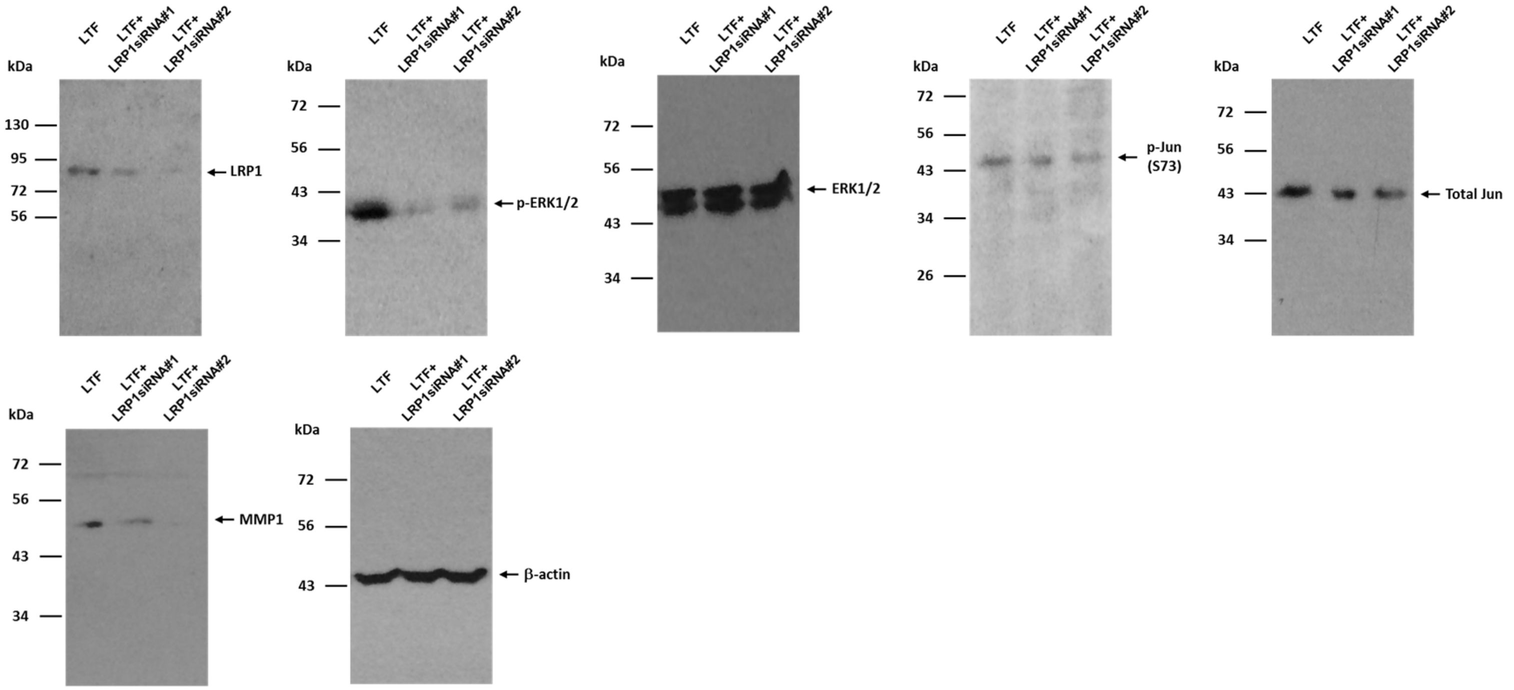

Fig. 6d

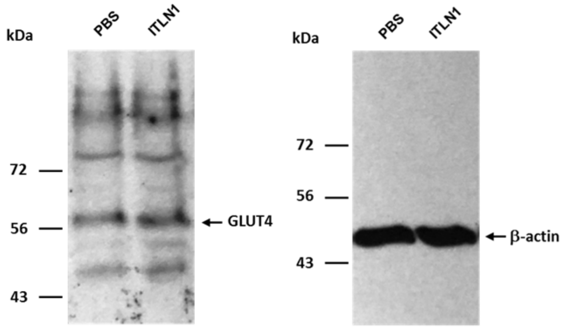

Supplementary Fig. 14: Original blots for Figs. 5c and 6.

Supplementary Fig. 15.

Supplementary Fig. 4d

SKOV3

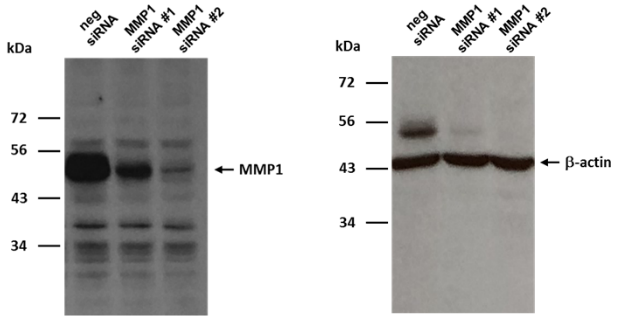

A224

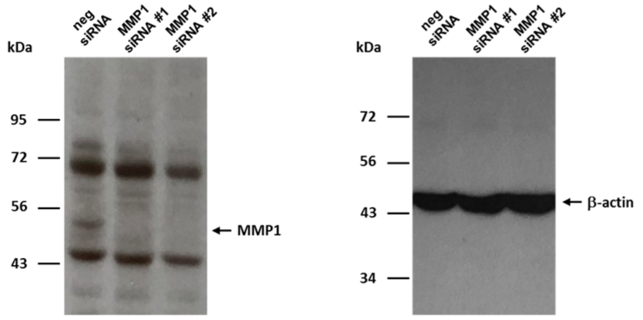

Supplementary Fig. 5b

SKOV3

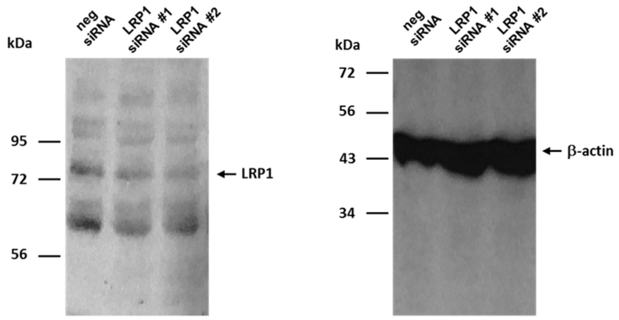

A224

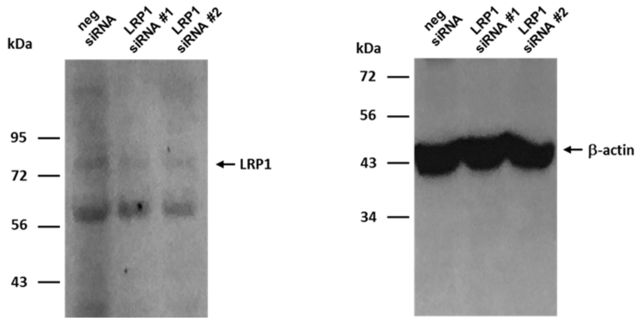

Supplementary Fig. 5c

SKOV3

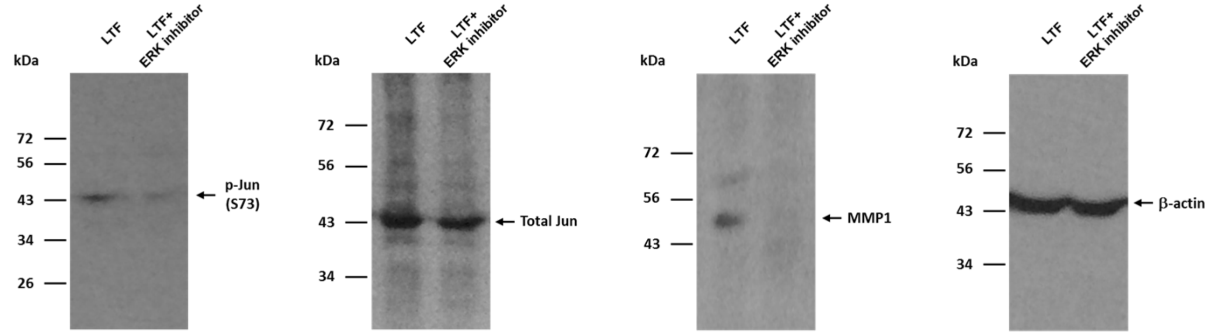

A224

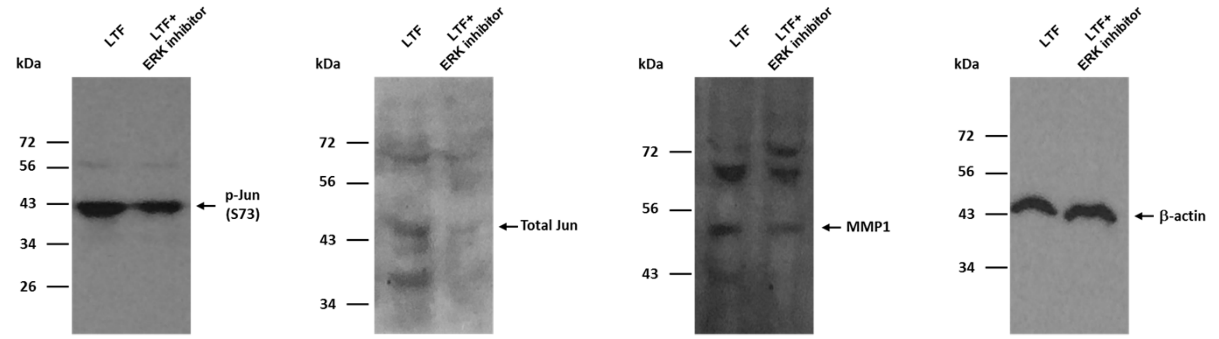

Supplementary Fig. 7c

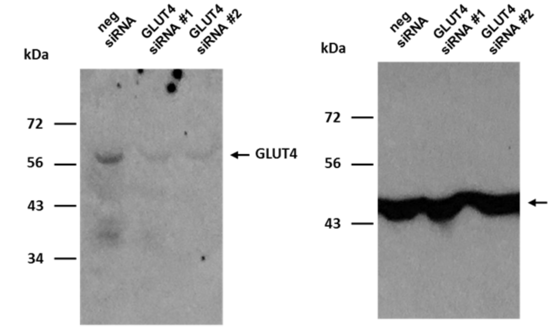

Supplementary Fig. 8b

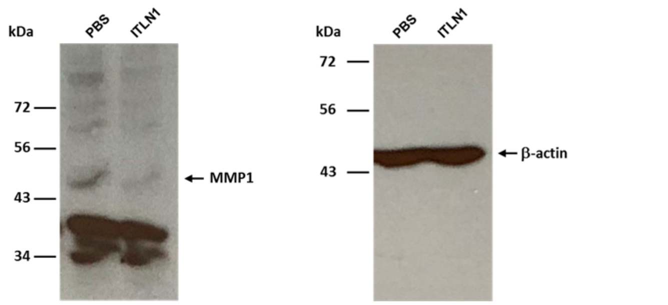

Supplementary Fig. 15: Original blots for Supplementary Figures.

Supplementary Table 1. Top down-regulated secretory protein-coded genes in cancer-associated mesothelial cells compared with those in normal mesothelial cells

Symbol	Entrez Gene Name	Log Fold Change	P-value*
ITLN1	Intelectin 1 (galactofuranose binding)	-6.675	3.50E-04
CPB1	Carboxypeptidase B1 (tissue)	-6.525	5.00E-05
SEPP1	Selenoprotein P, plasma, 1	-6.443	5.00E-05
KLK5	Kallikrein-related peptidase 5	-6.367	2.00E-04
ITIH3	Inter-alpha-trypsin inhibitor heavy chain 3	-6.338	1.00E+00
KLK7	Kallikrein-related peptidase 7	-5.648	5.00E-05
SLC27A2	Solute carrier family 27 (fatty acid transporter), member 2	-5.403	5.00E-05
IGFL2	IGF-like family member 2	-5.314	5.00E-05
FGF9	Fibroblast growth factor 9	-5.235	5.00E-05
SFTPD	Surfactant protein D	-5.172	5.00E-05
SERPINE3	Serpin peptidase inhibitor, clade E (nexin, plasminogen activator inhibitor type 1), member 3	-5.155	0.00005
HPD	4-hydroxyphenylpyruvate dioxygenase	-5.116	0.00005
THBD	Thrombomodulin	-5.047	0.00005
PRG4	Proteoglycan 4	-5.01	0.00005

* Two-tailed t-test

Supplementary Table 2. Expression levels of cytokines and their corresponding receptors in cancer-associated mesothelial cells compared with normal mesothelial cells

Gene	Entrez Gene Name	Fold Change	P-value*
**Cytokines**			
TNF	Tumor necrosis factor alpha	106.5073941	0.194407
TGFB1	Transforming growth factor beta 1	2.547404505	0.096925
TGFB2	Transforming growth factor beta 2	31.07795281	0.087245
**Cytokine receptors**			
TNFRSF1A	TNF receptor superfamily member 1A	1.37352688	0.206953
TNFRSF1B	TNF receptor superfamily member 1B	33.95832609	0.017973
TGFBR1	Transforming growth factor beta receptor I	0.806798319	0.450545
TGFBR2	Transforming growth factor beta receptor 2	2.204703167	0.003628
TGFBR3	Transforming growth factor beta receptor 3	1.603454962	0.187909

* Two-tailed t-test
