# Supplementary material for: ITLN1 modulates invasive potential and metabolic reprogramming of ovarian cancer cells in omental microenvironment
Source: Nat Commun. 2020 Jul 15;11:3546. doi: 10.1038/s41467-020-17383-2 (PMC7363861; doi:10.1038/s41467-020-17383-2)
Supplement: Supplementary file 3 — Reporting Summary [file 41467_2020_17383_MOESM3_ESM.pdf]

nature research

Corresponding author(s): Samuel C. Mok, Deepak Nagrath, Chi-Lam Au-Yeung

Last updated by author(s): 2020-6-17

# Reporting Summary

Nature Research wishes to improve the reproducibility of the work that we publish. This form provides structure for consistency and transparency in reporting. For further information on Nature Research policies, seeAuthors & Referees and theEditorial Policy Checklist .

## Statistics

For all statistical analyses, confirm that the following items are present in the figure legend, table legend, main text, or Methods section.

| n/a | Confirmed | |
|---|---|---|
| ☐ | ☒ | The exact sample size (*n*) for each experimental group/condition, given as a discrete number and unit of measurement |
| ☐ | ☒ | A statement on whether measurements were taken from distinct samples or whether the same sample was measured repeatedly |
| ☐ | ☒ | The statistical test(s) used AND whether they are one- or two-sided<br>*Only common tests should be described solely by name; describe more complex techniques in the Methods section.* |
| ☒ | ☐ | A description of all covariates tested |
| ☐ | ☒ | A description of any assumptions or corrections, such as tests of normality and adjustment for multiple comparisons |
| ☐ | ☒ | A full description of the statistical parameters including central tendency (e.g. means) or other basic estimates (e.g. regression coefficient) AND variation (e.g. standard deviation) or associated estimates of uncertainty (e.g. confidence intervals) |
| ☐ | ☒ | For null hypothesis testing, the test statistic (e.g. *F*, *t*, *r*) with confidence intervals, effect sizes, degrees of freedom and *P* value noted<br>*Give P values as exact values whenever suitable.* |
| ☒ | ☐ | For Bayesian analysis, information on the choice of priors and Markov chain Monte Carlo settings |
| ☒ | ☐ | For hierarchical and complex designs, identification of the appropriate level for tests and full reporting of outcomes |
| ☐ | ☒ | Estimates of effect sizes (e.g. Cohen's *d*, Pearson's *r*), indicating how they were calculated |

*Our web collection on statistics for biologists contains articles on many of the points above.*

## Software and code

Policy information about availability of computer code

| | |
|---|---|
| Data collection | IsoCor (LISBP), High Definition Imaging software (HDI1.4; Waters) |
| Data analysis | Progenesis QI (Waters), SPSS (23; IBM Corp.), R (3.4.0; The R Foundation) |

For manuscripts utilizing custom algorithms or software that are central to the research but not yet described in published literature, software must be made available to editors/reviewers. We strongly encourage code deposition in a community repository (e.g. GitHub). See the Nature Research guidelines for submitting code & software for further information.

## Data

Policy information about availability of data

All manuscripts must include a data availability statement. This statement should provide the following information, where applicable:

- Accession codes, unique identifiers, or web links for publicly available datasets
- A list of figures that have associated raw data
- A description of any restrictions on data availability

Data files from the transcriptome profiling analyses and RNA-sequencing analysis were deposited in the Gene Expression Omnibus Data Bank (GEO; National Cancer for Biotechnology Information, Bethesda, MD, USA). Transcriptome profiling data for microdissected normal and cancer-associated adipose tissue were under accession code GSE120196; RNA-sequencing data for normal and cancer-associated mesothelial cells were under accession code GSE120303 and transcriptome profiling data for ovarian cancer A224 cells with ITLN1 treatment were under accession code GSE120245.

# Field-specific reporting

Please select the one below that is the best fit for your research. If you are not sure, read the appropriate sections before making your selection.

# Life sciences study design

All studies must disclose on these points even when the disclosure is negative.

| | |
|---|---|
| Sample size | For in vitro studies, sample size was determined based on experience, variability of the results and previous literature. For in vivo studies, sample size was calculated based on the statistical software G*Power 3.1. Each experiment has a minimum power of 0.80 at alpha =0.05 to detect an overall group effect size of 1.2. |
| Data exclusions | No data was excluded from the analysis. |
| Replication | Three independent experiments were performed to verify the reproducibility of the experimental findings. All attempts were successful. |
| Randomization | For in vitro studies, cells were plated for all experimental groups at the same time and randomized into groups during treatment. For in vivo studies, mice were randomized into groups for different treatments after tumor establishment. |
| Blinding | Blinding is not relevant to our study. Data analyses were performed using the same pipeline/settings across groups to avoid bias. |

# Reporting for specific materials, systems and methods

We require information from authors about some types of materials, experimental systems and methods used in many studies. Here, indicate whether each material, system or method listed is relevant to your study. If you are not sure if a list item applies to your research, read the appropriate section before selecting a response.

## Materials & experimental systems

| n/a | Involved in the study | |
|---|---|---|
| ☐ | ☒ | Antibodies |
| ☐ | ☒ | Eukaryotic cell lines |
| ☒ | ☐ | Palaeontology |
| ☐ | ☒ | Animals and other organisms |
| ☐ | ☒ | Human research participants |
| ☒ | ☐ | Clinical data |

## Methods

| n/a | Involved in the study | |
|---|---|---|
| ☒ | ☐ | ChIP-seq |
| ☒ | ☐ | Flow cytometry |
| ☒ | ☐ | MRI-based neuroimaging |

# Antibodies

| | |
|---|---|
| Antibodies used | - The following primary antibodies were used for Western blotting: anti-ITLN1 (1:1,000; AB10627; EMD Millipore), anti-MMP1 (1:1,000; AB8105; EMD Millipore), anti-p-ERK1/2 (T202/204) (1:1,000; 9101; Cell Signaling Technology), anti-ERK1/2 (1:1,000; 9102; Cell Signaling Technology), anti-p-c-Jun (S73) (1:1,1000; 9164; Cell Signaling Technology), anti-c-Jun (1:1,000; 9165; Cell Signaling Technology), LRP-1 (1:1000; LS-B2675; LifeSpan BioSciences, Inc.), GLUT4 (1:1000; sc-1606; Santa Cruz Biotechnology Inc.), and b-actin (1:5,000; clone AC-15, Sigma-Aldrich Co.).<br>- The following primary antibodies were used for immunohistochemical analysis: anti-ITLN1 (1:500; A00020-01-100; Aviscera Bioscience Inc.) and anti-calretinin (1:500; NBP2-52426; Novus Biologicals) and anti-MMP1 (1:50; PA5-27210; Thermo Fisher Scientific).<br>- The following primary antibodies were used for Opal multiplex immunohistochemistry: anti-LTF (1:500, PA5-80784, Thermo Fisher Scientific), anti-CD11b (1:150, AC-0043RUO, Epitomics Inc.) and anti-CD66b (1:50, NB100-77808, Novus Biologicals).<br>- The following primary antibodies were used for Duolink PLA: anti-LRP-1 (1:200; ab92544; Abcam) and anti-LTF (1:100; sc-52048; Santa Cruz Biotechnology, Inc.).<br>-Horseradish peroxidase-conjugated secondary antibodies: Goat anti-Mouse IgG (H+L) Cross-Adsorbed Secondary Antibody, HRP (1:10000; 31432; Thermo Fisher Scientific), Goat anti-Rabbit IgG (H+L) Secondary Antibody, HRP (1:10000; 31460; Thermo Fisher Scientific), bovine anti-goat IgG-HRP (1:10000; sc-2350; Santa Cruz Biotechnology Inc.). |
| Validation | All antibodies used are commercially available. Based on the information from manufacturer's website, all of the following primary antibodies are specific for human origin, anti-ITLN1 (AB10627, EMD Millipore is specific for omentin in western blotting, anti-MMP1 (AB8105, EMD Millipore) recognizes an internal domain of human MMP-1 containing the hinge region. It does not cross react with MMP-2, MMP-9, or MMP-13; anti-p-ERK1/2 (T202/204) (9101, Cell Signaling Technology) detects endogenous levels of p44 and p42 MAP Kinase (Erk1 and Erk2) when phosphorylated either individually or dually at Thr202 and Tyr204 of Erk1 (Thr185 and Tyr187 of Erk2) in western blot. The antibody does not cross-react with the corresponding phosphorylated residues of either JNK/SAPK or p38 MAP Kinase, and does not cross-react with non-phosphorylated Erk1/2; anti-ERK1/2 (9102, Cell Signaling Technology) detects endogenous levels of total p44/42 MAP kinase (Erk1/Erk2) protein in western blot. The antibody does not recognize either JNK/SAPK or p38 MAP kinase; anti-p-c-Jun (S73) (9164, Cell Signaling Technology) detects endogenous levels of c-Jun only when activated by phosphorylation at Ser73 in western blot; anti-c-Jun monoclonal antibody (9165; Cell Signaling Technology) detects endogenous levels of total c-Jun protein, regardless of phosphorylation state, in western blot; LRP-1 (LS-B2675, LifeSpan BioSciences, Inc.) is validated in western blot; GLUT4 (sc-1606, Santa Cruz Biotecnology Inc.) detects epitope mapping within an N-terminal extracellular domain of Glut4 of human origin in western blot; b-actin is recommended for detection of b-Actin of broad species origin by WB including human; anti-ITLN1 (A00020-01-100, Aviscera Bioscience Inc.) detects omentin 1 on human visceral adipose tissues using immunohistochemistry; anti-calretinin (NBP2-52426, |

Novus Biologicals) detects purified recombinant fragment of human CALB2 (AA: 1-271) expressed in E. Coli and is recommended to use for immunohistochemistry; anti-MMP1 (PA5-27210, Thermo Fisher Scientific) is verified by relative expression to ensure that the antibody binds to the antigen stated; anti-LTF (PA5-80784, Thermo Fisher Scientific) has specificity for Human Lactotransferrin/LTF and is recommended for immunohistochemistry; anti-LRP-1 (ab92544, Abcam) is knockout validated and is recommended for immunofluorescence; anti-LTF (sc-52048, Santa Cruz Biotechnology, Inc.) is recommended for detection of lactoferrin of human origin by western blot and immunohistochemistry; anti-CD11b (AC-0043RUO, Epitomics Inc.) detects CD11b from human origin and is recommended to be used in immunohistochemistry; and anti-CD66b (NB100-77808, Novus Biologicals) detects CD66b antigen from human origin and is recommended to be used in immunohistochemistry.

## Eukaryotic cell lines

Policy information about cell lines

| | |
|---|---|
| Cell line source(s) | Human ovarian cancer lines A224 was a gift from Dr. Michael Birrer's laboratory at the Massachusetts General Hospital; SKOV3 was purchased from American Type Culture Collection, Manassas, VA, USA; OVCA432 and OVCA433, and mouse ovarian cancer line IG10 were gifts from Dr. Robert Bast's laboratory at MD Anderson Cancer Center. Primary normal mesothelial cells were isolated from omental adipose tissue from consented non-oncological adult donors undergoing elective gastric bypass surgery (Zen-Bio, Inc., Research Triangle Park, NC, USA) or from peritoneal washing from patients with benign gynecologic diseases. Primary cancer-associated mesothelial cells were cultured from the peritoneal effusions of HGSC patients. Mature adipocytes (mADSC) were differentiated from adipose-derived stem cells (ADSC) (American Type Culture Collection) under three cycles of differentiation. |
| Authentication | All cell lines were authenticated by short tandem repeat profiling in the Characterized Cell Line Core Facility at MD Anderson Cancer Center. |
| Mycoplasma contamination | All cell lines were tested negative for mycoplasma contamination. |
| Commonly misidentified lines (See ICLAC register) | There is no commonly misidentified cell lines used in the study. |

## Animals and other organisms

Policy information about studies involving animals; ARRIVE guidelines recommended for reporting animal research

| | |
|---|---|
| Laboratory animals | Female C57BL/6 mice at the age of 6 weeks old were used. Mice were maintained in barrier animal facilities approved by the American Association for Accreditation of Laboratory Animal Care (AAALAC) and in accordance with current regulations and standards of the United States Department of Agriculture (USDA), Department of Health and Human Services (DHHS), and National Institutes of Health (NIH). All Department of Veterinary Medicine and Surgery facilities' environmental conditions are monitored electronically by both Facilities Management and Edstrom Watchdog environmental monitoring system. Each room has designated temperature (ambient temperature) and humidity (40-60%) set-points and acceptable ranges. Lighting conditions (on and off) are also monitored. Animal room lights are controlled to turn on and off at designated times (12-h light/dark cycle). |
| Wild animals | No wild animals were used in the study. |
| Field-collected samples | No field-collected samples are used in the study. |
| Ethics oversight | The animal procedures were reviewed and approved by MD Anderson Cancer Center's institutional animal care and use committee. |

Note that full information on the approval of the study protocol must also be provided in the manuscript.

## Human research participants

Policy information about studies involving human research participants

| | |
|---|---|
| Population characteristics | All participants are females. Data collected include age, BMI, survival time, histology of tumors, stage of disease, and optimal or suboptimal surgery. |
| Recruitment | Healthy volunteer, or females being seen at MD Anderson Gynecologic Oncology outpatient clinic for suspected or confirmed caner, for any abnormality of the female organs that is not cancer (benign), or females having surgery to decrease their risk of developing cancer, also called "prophylactic surgery". |
| Ethics oversight | The samples from healthy women, patients with benign diseases or patients with HGSC were obtained from the ovarian cancer repository of the Department of Gynecologic Oncology and Reproductive Medicine under protocols approved by MD Anderson Cancer Center's institutional review board. |

Note that full information on the approval of the study protocol must also be provided in the manuscript.
